# The 2013 Ming K Jeang award for excellence in *Cell & Bioscience*

**DOI:** 10.1186/2045-3701-4-15

**Published:** 2014-03-24

**Authors:** Yun-Bo Shi

**Affiliations:** 1 The National Institutes of Health, Bethesda, MD, USA

## Abstract

Two research groups led by Yihong Ye of National Institute of Diabetes and Digestive and Kidney Diseases, National Institutes of Health, MD, USA and Dr. Lixin Wei of Medical Sciences Research Center, Renji hospital, School of Medicine, Shanghai Jiaotong University, Shanghai, China, won the 2013 Ming K Jeang Award for Excellence in *Cell & Bioscience.*

## Editorial

We are very pleased to announce that two research groups, who each published an outstanding research article in *Cell & Bioscience* in 2013, have been selected to receive the Ming K Jeang Award for Excellence in *Cell & Bioscience*. The Ming K Jeang Award for Excellence in *Cell & Bioscience* was established in 2011 with a generous donation from the Ming K. Jeang Foundation to honor outstanding research articles published in *Cell & Bioscience*, the official journal of the Society of Chinese Bioscientists in America (SCBA; http://www.scbasociety.org). A committee of *Cell & Bioscience* Editors, chaired by Dr. Chris Lau, considered all research articles published in the journal in 2013 to select the following two articles to receive the award [1,2].

Monoubiquitination of EEA1 regulates endosome fusion and trafficking.

Harish N Ramanathan, Guofeng Zhang, Yihong Ye

*Cell & Bioscience* 2013, **3**:24 (23 May 2013).

Autophagy lessens ischemic liver injury by reducing oxidative damage.

Kai Sun, Xuqin Xie, Yan Liu, Zhipeng Han, Xue Zhao, Ning Cai, Shanshan Zhang, Jianrui Song, Lixin Wei

*Cell & Bioscience* 2013, **3**:26 (10 June 2013).

Congratulations to these two groups of investigators for jobs well done!

We are looking forward to receiving contributions of outstanding research articles from the scientific community in 2014 and beyond.

## References

[B1] Ramanathan HN Zhang G Ye Y Monoubiquitination of EEA1 regulates endosome fusion and trafficking Cell & Bioscience 2013 3 24 10.1186/2045-3701-3-24 23701900 PMC3673817

[B2] Kai Sun K Xie X Liu Y Han Z Zhao X Cai N Zhang S Song J Wei L Autophagy lessens ischemic liver injury by reducing oxidative damage Cell & Bioscience 2013 3 26 10.1186/2045-3701-3-26 23758862 PMC3693965

